# Validation of 16S rRNA and Complete *rpoB* Gene Sequence Analysis for the Identification of *Elizabethkingia* Species

**DOI:** 10.3390/ijms241613007

**Published:** 2023-08-21

**Authors:** Jiun-Nong Lin, Chung-Hsu Lai, Chih-Hui Yang, Yi-Han Huang

**Affiliations:** 1 School of Medicine, College of Medicine, I-Shou University, Kaohsiung 824005, Taiwan; 2 Division of Infectious Diseases, Department of Internal Medicine, E-Da Hospital, I-Shou University, Kaohsiung 824005, Taiwan; 3 Department of Critical Care Medicine, E-Da Hospital, I-Shou University, Kaohsiung 824005, Taiwan; 4 Department of Biological Science and Technology, Meiho University, Pingtung 912009, Taiwan

**Keywords:** species identification, phylogenetic analysis, *Elizabethkingia miricola* cluster

## Abstract

Bacteria in the genus *Elizabethkingia* have emerged as a cause of life-threatening infections in humans. However, accurate species identification of these pathogens relies on molecular techniques. We aimed to evaluate the accuracy of 16S rRNA and complete RNA polymerase β-subunit (*rpoB*) gene sequences in identifying *Elizabethkingia* species. A total of 173 *Elizabethkingia* strains with whole-genome sequences in GenBank were included. The 16S rRNA gene and rpoB gene sequences from the same *Elizabethkingia* strains were examined. Of the 41 *E. meningoseptica* strains, all exhibited >99.5% 16S rRNA similarity to its type strain. Only 83% of the 99 *E. anophelis* strains shared >99.5% 16S rRNA gene similarity with its type strain. All strains of *E. meningoseptica* and *E. anophelis* formed a cluster distinct from the other *Elizabethkingia* species in the 16S rRNA and *rpoB* gene phylogenetic trees. The polymorphisms of 16S rRNA gene sequences are not sufficient for constructing a phylogenetic tree to discriminate species in the *E. miricola* cluster (*E. miricola*, *E. bruuniana*, *E. occulta*, and *E. ursingii*). The complete *rpoB* gene phylogenetic tree clearly delineates all strains of *Elizabethkingia* species. The complete *rpoB* gene sequencing could be a useful complementary phylogenetic marker for the accurate identification of *Elizabethkingia* species.

## 1. Introduction

Bacteria in the genus *Elizabethkingia* are aerobic Gram-negative bacilli distributed in the natural environments of water and soils and can contribute to infectious diseases in aquatic animals, such as fish and frogs [1,2]. Since their first isolation from infants with meningitis in 1959 [3], *Elizabethkingia* have sporadically been described to cause human infections [1]. Over the past ten years, however, these microorganisms have emerged as important opportunistic pathogens that result in a wide range of life-threatening infections in patients, including bloodstream infections, pneumonia, meningitis, biliary tract infection, and urinary tract infection [4]. The overall mortality rate of patients with *Elizabethkingia* infections is approximately 30% [4].

The taxonomy of species in the genus *Elizabethkingia* has undergone several modifications. Currently, seven validly named species have been described in this genus, namely *E. meningoseptica*, *E. anophelis*, *E. miricola*, *E. bruuniana*, *E. occulta*, *E. ursingii*, and *E. argenteiflava* (www.bacterio.net/elizabethkingia.html, accessed on 28 February 2023). Due to their close phylogenetic relationship, *E. miricola*, *E. bruuniana*, *E. occulta*, and *E. ursingii* are classified as the “*E. miricola* cluster”. Moreover, *E. anophelis* subsp. *endophytica*, previously proposed as a novel species *E. endophytica*, is now regarded as a subspecies rather than a separate species of the genus *Elizabethkingia* [5]. Conventional phenotypic methods such as biochemical tests and even matrix-assisted laser desorption ionization-time of flight mass spectrometry (MALDI-TOF MS) have been found to be challenging for accurately identifying all the species of the genus *Elizabethkingia* [1]. For example, almost all *E. anophelis* isolates were misidentified as *E. meningoseptica* using commercial microbial identification systems, such as API/ID32 Phenotyping Kits (v3.1, bioMérieux, Marcy l’Etoile, France), Phoenix 100 ID/AST Automated Microbiology System (v5.51A, Becton Dickinson Co., Sparks, MD, USA), VITEK 2 Automated Identification System (v7.01, bioMérieux, Marcy l’Etoile, France), VITEK MS MALDI-TOF MS System (v2.0/v3.0, bioMérieux, Marcy l’Etoile, France) [1]. The species-level identification of *Elizabethkingia* relies on genetics and molecular technology.

With the availability of high-throughput next-generation sequencing, whole-genome sequencing has been increasingly used for distinguishing bacterial species. In silico DNA–DNA hybridization (iDDH) based on whole-genome sequences can yield comparable, accurate, and precise results for bacterial species identification, similar to traditional DNA–DNA hybridization (DDH) [6,7]. However, the use of whole-genome sequencing for species delineation is usually limited to research purposes since this procedure remains complex, expensive, and time-consuming. Among the taxonomic markers for species identification, the 16S ribosomal RNA (rRNA) gene has been extensively used for bacterial phylogenetic analysis and species classification for decades [8]. However, bacteria contain multiple copies of the 16S rRNA gene, and intragenomic sequence variations between different copies have been reported in many microbes. The RNA polymerase β-subunit (*rpoB*) gene, a universally present housekeeping gene with a single copy, has been developed for bacterial phylogenetic analyses and molecular species identification [9].

Our previous study revealed that there are four or five copies of the 16S rRNA gene in *Elizabethkingia* species, and the intragenomic variation of the 16S rRNA gene sequences had no profound effect on the classification of taxa in most *Elizabethkingia* strains [10]. Although a few investigations have used the *rpoB* gene sequences in *Elizabethkingia* identification [11,12], the accuracy has never been comprehensively examined. The delineation of *Elizabethkingia* species using the 16S rRNA gene and *rpoB* gene sequences has not been thoroughly compared and studied. In the present study, we identified the precise *Elizabethkingia* species of strains with whole-genome sequences available in the GenBank of the National Center for Biotechnology Information (NCBI) genome sequence repository using the iDDH analyses. We then investigated the accuracy of sequence similarity and the phylogenetic tree derived from the 16S rRNA gene and complete *rpoB* gene for the identification of *Elizabethkingia* species.

## 2. Results

### 2.1. Nucleotide Polymorphisms in the 16S rRNA and rpoB Gene

**Figure 1:**
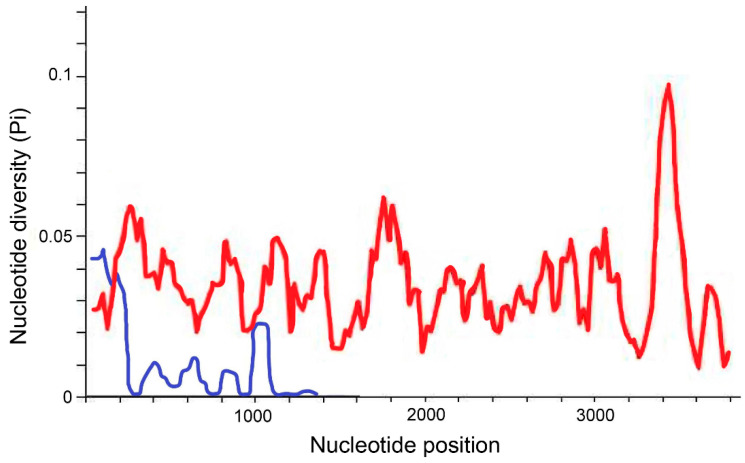
Nucleotide polymorphisms in the 16S rRNA gene sequences (blue line) and *rpoB* gene sequences (red line) of 173 *Elizabethkingia* strains included in this study. The x-axis indicates the positions of nucleotides, and the y-axis indicates the nucleotide diversity (Pi) in windows of 100 nucleotides.

At the time of study preparation in October 2022, there were 173 whole-genome sequences of *Elizabethkingia* species available in GenBank (Table S1). Figure 1 shows the nucleotide variations of the 1517-bp long 16S rRNA gene and 3825-bp long *rpoB* gene in these 173 *Elizabethkingia* strains. Nucleotide polymorphisms in the 16S rRNA genes were most frequent at positions 100–200 and 1000–1100. For the *rpoB* genes, the most hypervariable region was at positions 3300–3500, followed by positions 1700–1800 and 200–300. The 16S rRNA gene had 83 polymorphic sites, while the *rpoB* gene had 809 polymorphic sites. The degree of polymorphism was lower in the 16S rRNA gene (nucleotide diversity, Pi: 0.00969) compared to the *rpoB* gene (nucleotide diversity, Pi: 0.03452).

### 2.2. Species Identification Based on Whole-Genome Sequences

In the present study, species identification using iDDH analyses yielded consistent results for the 173 *Elizabethkingia* strains tested. Based on whole-genome analysis, 99 strains were identified as *E. anophelis*, 41 as *E. meningoseptica*, 16 as *E. miricola*, 10 as *E. bruuniana*, four as *E. ursingii*, two as *E. occulta*, and one as *E. argenteiflava* (Table S1). Notably, *E. miricola* strain EM_CHUV had the lowest iDDH value (72.42%).

### 2.3. Similarity of 16S rRNA Gene and rpoB Gene Sequences among Type Strains

The similarity of the 16S rRNA sequence among the type strains *E. anophelis* R26, *E. meningoseptica* KC1913, and *E. argenteiflava* YB22 was less than 99.5% (the pink part of Table 1). However, type strains *E. miricola*, *E. bruuniana*, *E. occulta*, and *E. ursingii* shared over 99.5% 16S rRNA gene sequence identity with each other. For example, *E. miricola* GTC 862 showed a 99.86% sequence identity to *E. bruuniana* G0146, a 99.52% identity to *E. ursingii* G4122, and a 99.59% identity to *E. occulta* G4070. The 16S rRNA sequence identity between *E. bruuniana* G0146 and *E. occulta* G4070 was 99.54%.

Regarding the *rpoB* gene sequence, *E. meningoseptica* KC1913, *E. anophelis* R26, and *E. argenteiflava* YB22 demonstrated a sequence similarity of less than 97.7% to each other (the green part of Table 1). However, *E. miricola* GTC 862 displayed a *rpoB* sequence identity of 99.14% to *E. bruuniana* G0146, 98.85% identity to *E. ursingii* G4122, and 98.17% identity to *E. occulta* G4070. The *rpoB* sequence identity between *E. bruuniana* G0146 and *E. ursingii* G4122, *E. bruuniana* G0146 and *E. occulta* G4070, and *E. ursingii* G4122 and *E. occulta* G4070 was 98.77%, 98.22%, and 98.38%, respectively.

### 2.4. Species Identification of E. anophelis Using 16S rRNA Gene and rpoB Gene Sequencing

**Figure 2:**
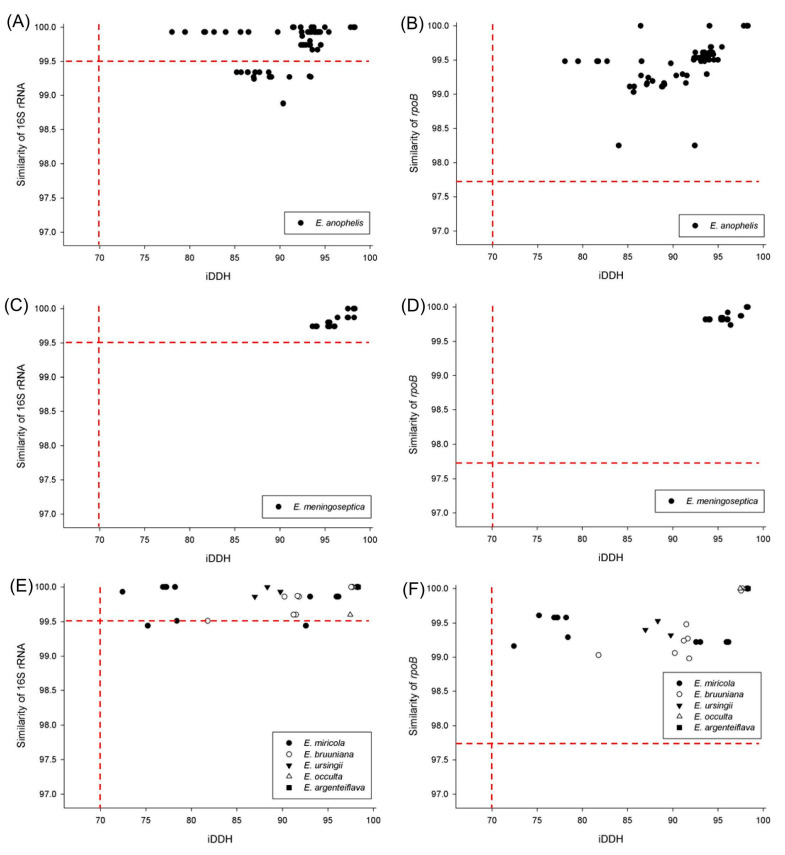
Scatter plot of the relationship between in silico DNA–DNA hybridization (iDDH) values and sequence similarities of the 16S rRNA gene (**left** parts) and sequence similarities of the complete *rpoB* gene (**right** parts) in 173 *Elizabethkingia* strains. (**A**,**B**) 99 *E. anophelis* strains. (**C**,**D**) 41 *E. meningoseptica* strains. (**E**,**F**) 16 *E. miricola* strains, 10 *E. bruuniana* strains, 4 *E. ursingii* strains, 2 *E. occulta* strains, and 1 *E. argenteiflava* strain.

Among the 99 strains of *E. anophelis*, 82 strains shared over 99.5% 16S rRNA gene sequence identity with *E. anophelis* type strain R26; the remaining 17 strains shared only 98.88–99.34% sequence identity (Figure 2A and Table S1). Out of these 17 strains, 14 had over 99.5% 16S rRNA gene sequence identity to *E. anophelis* subsp. *endophytica* type strain JM-87 (Table S2), but three strains (*E. anophelis* CIP60.58, *E. anophelis* PW2809, and *E. anophelis* SEA01) had a sequence identity of 98.29–99.34% to *E. anophelis* subsp. *endophytica* JM-87. It is worth noting that *E. anophelis* SEA01 exhibited less than 99% 16S rRNA sequence similarity to *E. anophelis* type strain R26 and *E. anophelis* subsp. *endophytica* type strain JM-87. All strains of *E. anophelis* formed a distinct group in the 16S rRNA gene phylogenetic tree (Figure S1).

The complete *rpoB* sequences of the 99 *E. anophelis* strains shared 98.25–100% similarity with *E. anophelis* type strain R26 (Figure 2B and Table S1). All *E. anophelis* strains formed a separate cluster in the complete *rpoB* phylogenetic tree, distinct from other *Elizabethkingia* species (Figure S1).

### 2.5. Species Identification of E. meningoseptica Using 16S rRNA Gene and rpoB Gene Sequencing

All 41 *E. meningoseptica* strains shared 99.74–100% 16S rRNA gene sequence similarity with *E. meningoseptica* type strain KC1913 (Figure 2C and Table S1). The phylogenetic tree based on the 16S rRNA sequence showed that all strains of *E. meningoseptica* clustered together, distinct from the other six *Elizabethkingia* species (Figure S1). Regarding the complete *rpoB* sequence, the 41 *E. meningoseptica* strains demonstrated a 99.74–100% sequence similarity to *E. meningoseptica* type strain KC1913 (Figure 2D and Table S1). The *rpoB* gene phylogenetic trees showed that the 41 *E. meningoseptica* strains formed a distinct cluster separate from other *Elizabethkingia* species (Figure S1).

### 2.6. Species Identification of E. miricola Cluster Strains (E. miricola, E. bruuniana, E. occulta, and E. ursingii) Using 16S rRNA Gene and rpoB Gene Sequencing

The sequence similarity of the 16S rRNA and complete *rpoB* genes among strains of the *E. miricola* cluster is shown in Table 2. The sequence similarity provides poor discriminatory power for the taxonomic classification of these *Elizabethkingia* species. Two *E. miricola* strains and one *E. bruuniana* strain had less than 99.5% 16S rRNA sequence similarity to their respective type strains (Figure 2E). All strains in the *E. miricola* cluster shared over 97.7% *rpoB* sequence similarity with their type strains (Figure 2F). However, among the 16 *E. miricola* strains, three had the highest 16S rRNA sequence identity with *E. ursingii* type strain G4122, and one exhibited the highest *rpoB* sequence similarity to *E. bruuniana* type strain G0146. Out of the ten *E. bruuniana* strains, four shared the highest 16S rRNA sequence similarity with *E. miricola* type strain GTC 862, and four had *rpoB* sequences most similar to *E. ursingii* type strain G4122. Notably, *E. miricola* strain EM_CHUV and *E. bruuniana* strain CSID 3015183685 are extreme examples where the 16S rRNA sequences are completely identical to those of other type strains.

**Figure 3:**
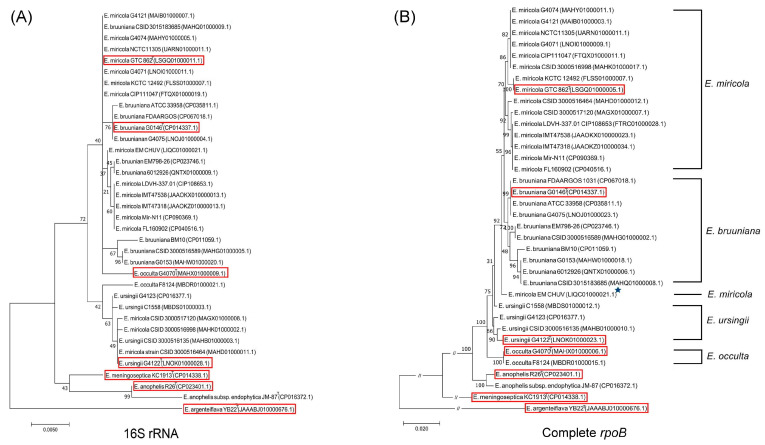
Phylogenetic analysis derived from the 16S rRNA gene sequences (**A**) and complete *rpoB* gene sequences (**B**) in *E. miricola* cluster strains (*E. miricola*, *E. bruuniana*, *E. occulta*, and *E. ursingii*) using the maximum-likelihood method based on the Jukes–Cantor model (JC69). The percentage of replicate trees in which the associated taxa clustered together in the bootstrap test of 1000 replicates and branch lengths of the evolutionary distances are shown. The asterisk represents the strain with unexpected positioning in the tree. Type strains are marked in red windows.

**Figure 4:**
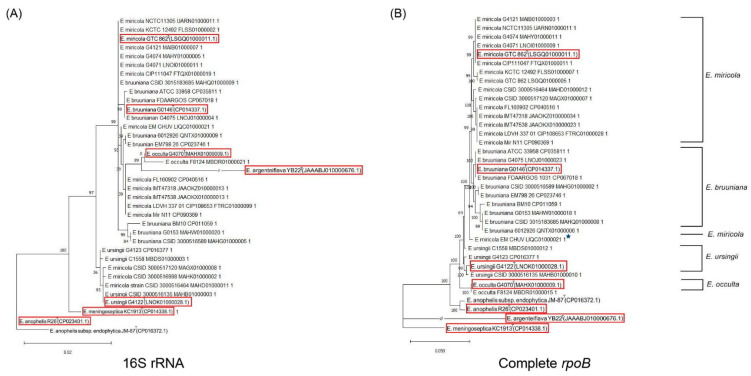
Phylogenetic analysis derived from the 16S rRNA gene sequences (**A**) and complete *rpoB* gene sequences (**B**) in *E. miricola* cluster strains (*E. miricola*, *E. bruuniana*, *E. occulta*, and *E. ursingii*) using the approximate likelihood-ratio test with Bayes branch support based on the Jukes–Cantor model (JC69). The percentages of replicate trees are computed along PhyML on the original data set. The branch support is significant if it is >0.90. The asterisk represents the strain with unexpected positioning in the tree. Type strains are marked in red windows.

The phylogenetic tree constructed based on the 16S rRNA gene revealed that strains of *E. miricola*, *E. bruuniana*, *E. ursingii*, and *E. occulta* formed a close cluster (Figure S1). Some strains were located at ambiguous or fallacious positions in this 16S rRNA tree (Figure 3). For example, *E. bruuniana* strain CSID 3015183685, *E. bruuniana* strain EM798-26, and *E. bruuniana* strain 6012926 were located within groups *E. miricola*. *E. occulta* strain F8124, *E. miricola* strain CSID 3000516998, *E. miricola* strain CSID 3000517120, and *E. miricola* strain CSID 3000516464 and were most closely related to *E. ursingii* type strain G4122. The *rpoB* gene sequence-based phylogenetic tree provided a clear classification of *Elizabethkingia* species for all tested strains except the *E. miricola* strain EM_CHUV (Figure 3). Compared to the 16S rRNA phylogenetic tree, the analysis based on *rpoB* gene sequences provided a more reliable identification of *Elizabethkingia* species supported by higher bootstrap values. The Bayesian phylogenetic analysis also showed that a lot of *Elizabethkingia* strains were located at unexpected positions in the tree based on the 16S rRNA gene sequences, and the *rpoB* gene sequence tree revealed a clear classification of all *Elizabethkingia* strains except the *E. miricola* strain EM_CHUV (Figure 4). The values of Bayes branch support calculated using approximate likelihood-ratio tests in the phylogenetic tree based on *rpoB* gene sequences were higher than those in the tree constructed based on the 16S rRNA gene sequences.

## 3. Discussion

Sequencing of the 16S rRNA gene has been extensively used for bacterial identification and taxonomic classification. The 16S rRNA gene consists of nine hypervariable regions (V1–V9) [10]. In our previous study [10], we found that intraspecific heterogeneity is most commonly observed in the hypervariable regions V2 (nucleotide positions 137–242) and V6 (positions 986–1043), which is consistent with the findings of the present study. The entire 16S rRNA gene can be easily sequenced using the Sanger method with two amplification primers and five sequencing primers [13,14]. Since partial sequencing of the 16S rRNA gene usually provides limited discriminatory power for bacterial species classification, sequencing of the full-length 16S rRNA gene is recommended regardless of the distribution of the hypervariable regions [11,13,14].

The *rpoB* gene has a total length of 3825 bp, and sequencing the entire gene requires more primers compared to the 16S rRNA gene when using the Sanger method. Several studies have suggested that the hypervariable region for species identification in many bacteria lies between positions 2300 and 3300 of the *rpoB* gene [8,15,16]. In line with these findings, our study revealed that the most hypervariable region of the *rpoB* gene is located between positions 3300 and 3500, which aligns with previous observations in other bacteria [8,15,16].

Previous studies have reported that isolates with less than 99% 16S rRNA gene sequence identity often exhibit less than 70% DDH (DNA–DNA hybridization) values with their type strains, indicating that they belong to different species [17]. However, isolates with more than 99% 16S rRNA gene sequence similarity may not share more than 70% DDH with the type strain and are still classified within the same species [8]. Janda et al. [18] proposed a minimum threshold of >99% 16S rRNA gene sequence similarity for species identification, with an ideal threshold of >99.5%. However, the 16S rRNA sequence has been found to have limited phylogenetic and discriminatory power for bacterial classification in certain bacteria, such as *Aeromonas*, *Enterobacter*, rapid-growing mycobacteria, and *Acinetobacter* [18]. In our present study, all strains of *E. meningoseptica* exhibited 16S rRNA gene similarity values above the >99.5% cutoff, and they formed a distinct cluster in the 16S rRNA phylogenetic tree. However, among the 99 *E. anophelis* strains, only 83% of strains shared >99.5% 16S rRNA gene similarity with the *E. anophelis* type strain R26, and one strain notably exhibited less than 99% 16S rRNA sequence identity to the type strain. Nevertheless, all *E. anophelis* strains formed a separate cluster from other *Elizabethkingia* species in the 16S rRNA phylogenetic trees. Therefore, relying solely on the criteria of >99.5% 16S rRNA sequence similarity to the type strain is not sufficient for the identification of *E. anophelis*. Analysis of 16S rRNA phylogenetic relationships is necessary for the delineation of *E. anophelis* strains.

The similarity of the *rpoB* gene sequence can accurately reflect the degree of DNA reassociation between the tested bacteria [15,19]. However, the cutoff value for *rpoB* gene sequence similarity depends on the length of the sequence fragment [8]. For instance, the suggested sequence identity criteria for discriminating at the species level for fragments of 300–600 bp, 600–825 bp, and the full length of the *rpoB* gene are 94–95%, 96–97%, and 97.7%, respectively [8]. In our present study, the similarity of complete *rpoB* gene sequences among *E. anophelis* strains and its type strain ranged from 98.25% to 100%, while for *E. meningoseptica* strains, it was 99.74% to 100%. All strains of *E. anophelis* and *E. meningoseptica* also clustered together in their corresponding groups in the complete *rpoB* gene-based phylogenetic tree.

Our study revealed that sequencing of the 16S rRNA gene is apparently insufficient to provide adequate delimitation power for these closely related species, including *E. miricola*, *E. bruuniana*, *E. occulta*, and *E. ursingii*. Several strains in the *E. miricola* cluster shared >99.5% 16S rRNA gene sequence similarity. Out of the 32 strains in the *E. miricola* cluster, 21.9% exhibited the highest identity rate with other type strains, and two strains possessed completely matching 16S rRNA sequences to other type strains. Moreover, the polymorphisms of the 16S rRNA gene sequences are not satisfactory for constructing a phylogenetic tree to differentiate *E. miricola*, *E. bruuniana*, *E. occulta*, and *E. ursingii*. Therefore, even if the 16S rRNA sequences are nearly or completely identical to type strains, additional molecular methods are still necessary to accurately identify the species within the *E. miricola* cluster strains.

Sequencing the *rpoB* gene has been shown to be a complementary tool to 16S rRNA sequencing for species identification and phylogenetic analyses in certain bacteria [8,14]. Our study revealed that five out of 32 strains (15.6%) in the *E. miricola* cluster exhibited the highest *rpoB* sequence similarity to other type strains instead of their corresponding type strain. Therefore, species identification of strains in the *E. miricola* cluster cannot solely rely on the comparison of *rpoB* gene sequence similarity. The analysis of the phylogenetic tree is necessary to effectively distinguish different clusters of *E. miricola*, *E. bruuniana*, *E. occulta*, and *E. ursingii.*

## 4. Materials and Methods

### 4.1. Study Design

This study searched the GenBank database (https://www.ncbi.nlm.nih.gov/genome/, accessed on 8 December 2022) to find *Elizabethkingia* strains with submitted whole-genome sequences. The non-duplicate whole-genome sequences were downloaded for analysis. The complete 16S rRNA gene sequence and complete *rpoB* gene sequences from the same *Elizabethkingia* strains were inspected to constitute the database for this study. 

### 4.2. Species Identification Based on Whole-Genome Sequences Analysis

The accurate species of the *Elizabethkingia* strains with submitted whole-genome sequences in GenBank were identified using iDDH analyses. The iDDH values were calculated using the Genome-to-Genome Distance Calculator v3.0, and an iDDH cutoff value of 70% was used as the species delimitation criterion [7].

### 4.3. Sequence Analysis and Phylogenetic Tree Construction

The sequences were aligned using ClustalW v2.1 with the default options in MEGA v11.0.13. (https://www.megasoftware.net/, accessed on 8 August 2023). The similarity between sequences was calculated using the NCBI blastn suite (https://blast.ncbi.nlm.nih.gov/Blast.cgi, accessed on 28 February 2023). The degrees of nucleotide variability in the entire 16S rRNA gene sequence (1517 bp) and complete *rpoB* gene sequence (3825 bp) among the different strains were calculated using the DnaSP v.6.12.01 software [20]. A minimum criterion for species identification is a 16S rRNA sequence with 99% similarity to the type strain, while >99.5% similarity is considered ideal for species delineation [14]. A cutoff value of >97.7% similarity for the complete *rpoB* gene sequence is suggested as a criterion for species boundary [8]. Phylogenetic trees of the gene sequences were constructed using the maximum likelihood method based on the Jukes–Cantor model (JC69) with 1000 bootstrap replications using MEGA v11.0.13. The Bayesian phylogenetic analysis was completed using the DIVEIN web server [21].

## 5. Conclusions

The results of this study demonstrate that combining 16S rRNA sequence similarity comparison with phylogenetic tree analysis can accurately identify all strains of *E. anophelis* and *E. meningoseptica*. However, it is unable to accurately distinguish species within the *E. miricola*, *E. bruuniana*, *E. occulta*, and *E. ursingii* clusters. The analysis of the complete *rpoB* gene sequence is still necessary to avoid erroneously identifying the species of *E. miricola*, *E. bruuniana*, *E. occulta*, and *E. ursingii*.

## Figures and Tables

**Table 1:** Similarity of the 16S rRNA sequence (the pink part) and *rpoB* sequence (the green part) between type strains of *Elizabethkingia* species.

	*E. anophelis* R26	*E. meningoseptica* KC1913	*E. miricola* GTC 862	*E. bruuniana* G0146	*E. ursingii* G4122	*E. occulta* G4070	*E. argenteiflava* YB22
*E. anophelis* R26	100	93.05	97.65	97.49	97.59	97.25	84.22
*E. meningoseptica* KC1913	98.82	100	93.05	92.89	93.02	92.76	84.19
*E. miricola* GTC 862	98.69	98.76	100	99.14	98.85	98.17	83.77
*E. bruuniana* G0146	98.62	98.68	99.86	100	98.77	98.22	83.62
*E. ursingii* G4122	98.49	99.08	99.52	99.47	100	98.38	83.72
*E. occulta* G4070	98.35	98.42	99.59	99.54	99.28	100	83.56
*E. argenteiflava* YB22	96.65	97.04	96.77	96.91	96.77	97.1	100

*E. anophelis* R26 (GenBank accession no. CP014337.1); *E. meningoseptica* KC1913 (GenBank accession no. CP035809.1); *E. miricola* GTC 862 (GenBank accession no. LSGQ01000005.1); *E. bruuniana* G0146 (GenBank accession no. CP014337.1); *E. ursingii* G4122 (GenBank accession no. LNOK01000023.1); *E. occulta* G4070 (GenBank accession no. MAHX01000006.1); *E. argenteiflava* YB22 (GenBank accession no. JAAABJ010000676.1).

**Table 2:** Sequence similarity of the 16S rRNA gene (middle columns) and *rpoB* gene (right columns) to type strains *E. miricola*, *E. bruuniana*, *E. occulta*, and *E. ursingii*.

Strain (Accession no.)	16S rRNA					*rpoB*			
	*E. miricola* GTC 862	*E. bruuniana* G0146	*E. ursingii* G4122	*E. occulta* G4070		*E. miricola* GTC 862	*E. bruuniana* G0146	*E. ursingii* G4122	*E. occulta* G4070
*E. miricola* CIP111047 (FTQX01000011.1)	100	99.87	99.51	99.6		99.82	99.35	98.99	98.34
*E. miricola* LDVH-337.01 (CIP108653.1)	99.86	99.8	99.51	99.6		99.29	99.23	98.88	98.34
*E. miricola* IMT47538 (JAAOKX010000023.1)	99.86	99.8	99.51	99.6		99.29	99.23	98.88	98.34
*E. miricola* IMT47318 (JAAOKZ010000034.1)	99.86	99.8	99.51	99.6		99.29	99.23	98.88	98.34
*E. miricola* KCTC 12492 (FLSS01000007.1) (type strain) ^a^	100	99.87	99.51	99.6		100	99.23	98.88	98.17
*E. miricola* EM CHUV (LIQC01000021.1)	99.93	99.8	99.44	99.54		99.29	99.35	99.11	98.11
*E. miricola* G4071 (LNOI01000009.1)	100	99.87	99.51	99.6		99.82	99.35	98.99	98.34
*E. miricola* GTC 862 (LSGQ01000005.1) (type strain) ^a^	100	99.87	99.51	99.6		100	99.23	98.88	98.17
*E. miricola* CSID 3000517120 (MAGX01000007.1)	99.44	99.41	99.93	99.21		99.29	99.23	98.88	98.34
*E. miricola* CSID 3000516464 (MAHD01000012.1)	99.51	99.47	100	99.28		99.35	99.29	98.94	98.29
*E. miricola* CSID 3000516998 (MAHK01000017.1)	99.44	99.41	99.93	99.21		99.88	99.29	98.94	98.29
*E. miricola* NCTC11305 (UARN01000011.1)	100	99.87	99.51	99.6		99.82	99.35	98.99	98.34
*E. miricola* G4074 (MAHY01000011.1)	100	99.87	99.51	99.6		99.82	99.35	98.99	98.34
*E. miricola* Mir-N11 (CP090369.1)	99.86	99.8	99.44	99.6		99.29	99.23	98.88	98.34
*E. miricola* G4121 (MAIB01000003.1)	100	99.87	99.51	99.6		99.82	99.35	98.99	98.34
*E. miricola* FL160902 (CP040516.1)	99.86	99.8	99.51	99.6		99.29	99.23	98.88	98.34
*E. bruuniana* G0146 (CP014337.1) (type strain)	99.86	100	99.44	99.54		99.23	100	98.94	98.17
*E. bruuniana* BM10 (CP011059.1)	99.51	99.47	99.24	99.27		99.05	99.11	99.23	97.99
*E. bruuniana* EM798-26 (CP023746.1)	99.86	99.87	99.51	99.54		99.11	99.53	98.94	98.17
*E. bruuniana* 6012926 (QNTX01000006.1)	99.86	99.87	99.44	99.54		98.99	99.05	99.17	97.93
*E. bruuniana* CSID 3000516589 (MAHG01000002.1)	99.72	99.6	99.24	99.34		99.11	99.53	98.94	98.17
*E. bruuniana* CSID 3015183685 (MAHQ01000008.1)	100	99.87	99.51	99.6		99.05	99.11	99.23	97.99
*E. bruuniana* FDAARGOS 1031 (CP067018.1)	99.86	100	99.44	99.54		99.23	100	98.94	98.17
*E. bruuniana* G0153 (MAHW01000018.1)	99.72	99.6	99.24	99.34		98.99	99.05	99.17	97.93
*E. bruuniana* ATCC 33958 (CP035811.1)	99.86	100	99.44	99.54		99.23	100	98.94	98.17
*E. bruuniana* G4075 (LNOJ01000023.1)	99.86	100	99.44	99.54		99.23	100	98.94	98.17
*E. ursingii* G4122 (LNOK01000023.1) (type strain)	99.51	99.47	100	99.28		98.88	98.94	100	98.4
*E. ursingii* G4123 (CP016377.1)	99.58	99.47	99.93	99.21		98.58	98.76	99.47	98.46
*E. ursingii* CSID 3000516135 (MAHB01000010.1)	99.51	99.47	100	99.28		98.64	98.82	99.53	98.64
*E. ursingii* C1558 (MBDS01000012.1)	99.51	99.41	99.86	99.28		98.94	99.11	99.47	98.46
*E. occulta* G4070 (MAHX01000006.1) (type strain)	99.58	99.54	99.24	100		98.17	98.17	98.4	100
*E. occulta* F8124 (MBDR01000015.1)	99.17	99.14	99.51	99.6		98.17	98.17	98.4	100

^a^ *E. miricola* type strain KCTC 12492 = *E. miricola* type strain GTC 862.

## Data Availability

All genome sequences used in this study were downloaded from the GenBank database (https://www.ncbi.nlm.nih.gov/genome/). The GenBank accession numbers of the DNA sequences used in the present study are available in Table S1 in the supplemental material.

## Supplementary Materials

The following supporting information can be downloaded at: https://www.mdpi.com/article/10.3390/ijms241613007/s1.
