# Supplementary material for: Validation of 16S rRNA and Complete rpoB Gene Sequence Analysis for the Identification of Elizabethkingia Species
Source: Int J Mol Sci. 2023 Aug 21;24(16):13007. doi: 10.3390/ijms241613007 (PMC10455528; doi:10.3390/ijms241613007)
Supplement: Supplementary file 1 [file ijms-24-13007-s001.zip › ijms-2522072-supplementary.pdf]

Table S1. *Elizabethkingia* strains, isolation source, isolation country, iDDH value, and similarity of 16S rRNA gene and complete *rpoB* gene to each type strain.

Strain (GenBank accession no.)	Isolation source	Isolation country	iDDH value	16S rRNA similarity (%)	Complete *rpoB* similarity (%)
E. anophelis 502 (AVCQ01000002.1)	Wound swab	United Kingdom	91.07	99.27	99.29
E. anophelis CIP60.58 (FTQY01000009.1)	NA	NA	93.42	99.27	99.56
E. anophelis CIP111046 (FTRB01000010.1)	NA	NA	87.09	99.27	99.14
E. anophelis CIP111067 (FTQZ01000016.1)	NA	NA	92.48	99.87	99.61
E. anophelis E18064 (CCAB010000143.1)	NA	NA	92.41	99.93	98.25
E. anophelis DSM-23781 (FLST01000010.1)	NA	NA	98.21	100	100
E. anophelis PW2810 (CBYF010000021.1)	NA	NA	88.85	99.27	99.11
E. anophelis NUH1 (ASYH01000018.1)	Hand hygiene sink aerator	Singapore	81.59	99.93	99.48
E. anophelis NUH4 (ASYI01000018.1)	Hand hygiene sink aerator	Singapore	79.48	99.93	99.48
E. anophelis NUH6 (ASYJ01000035.1)	Hand hygiene sink aerator	Singapore	86.54	99.93	99.48
E. anophelis NUH11 (ASYK01000059.1)	Hand hygiene sink aerator	Singapore	86.52	99.93	99.48
E. anophelis NUHP2 (ASYF01000012.1)	Hand hygiene sink aerator	Singapore	81.72	99.93	99.48
E. anophelis NUHP3 (ASYG01000015.1)	NA	Singapore	81.72	99.93	99.48
E. anophelis PW2806 (CBYD010000040.1)	NA	NA	93.33	99.93	99.56
E. anophelis PW2809 (CBYE010000030.1)	NA	NA	93.29	99.28	99.56
E. anophelis 3375 (MAHM01000015.1)	NA	USA	92.37	99.74	99.53
E. anophelis 6499925 (QNTZ01000010.1)	Blood	USA	93.32	99.74	99.61
E. anophelis 40313151 (QNTY01000011.1)	Blood	USA	92.61	99.74	99.53
E. anophelis B2D (JNCG01000001.1)	Dental plaque	Malaysia	85.21	99.34	99.11

E. anophelis BP8467 (VTFI01000009.1)	Blood	Vellore	93.34	99.8	99.61
E. anophelis CSID 3000516074 (MAHA01000005.1)	NA	USA	92.35	99.74	99.53
E. anophelis CSID 3000516810 (MAHH01000024.1)	NA	USA	93.4	99.93	99.5
E. anophelis CSID 3000516978 (MAHJ01000016.1)	NA	USA	87.26	99.34	99.24
E. anophelis CSID 3000517066 (MAHL01000016.1)	NA	USA	86.46	99.34	99.27
E. anophelis CSID 3015183679 (MAHO01000015.1)	NA	USA	89.76	99.93	99.45
E. anophelis EM361-97 (LWDS01000010.1)	Blood	Taiwan	91.43	100	99.16
E. anophelis Po0527107 (CCAC010000034.1)	NA	NA	92.42	99.93	98.25
E. anophelis LDVH-AR107 (CIP108654.1)	Internal organs	France	83.99	99.93	98.25
E. anophelis SQ122 (JADRJG010000001.1)	Blood	China	93.87	99.93	99.53
E. anophelis EA1 (JACLEZ010000013.1)	NA	China	85.64	99.93	99.03
E. anophelis EA2 (JACLFD010000007.1)	NA	China	93.28	99.74	99.53
E. anophelis EA3 (JACLFE010000013.1)	NA	China	85.64	99.93	99.03
E. anophelis EA4 (JACLFF010000007.1)	NA	China	94.05	99.93	99.61
E. anophelis EA5 (JACLFG010000007.1)	NA	China	94.05	99.93	99.61
E. anophelis EA6 (JACLFH010000017.1)	NA	China	97.84	100	100
E. anophelis EA7 (JACLFI010000009.1)	NA	China	94.38	99.93	99.61
E. anophelis EA8 (JACLFJ010000009.1)	NA	China	94.38	99.93	99.61
E. anophelis EA9 (JACLFK010000006.1)	NA	China	94.09	99.93	99.61
E. anophelis EA10 (JACLEP010000007.1)	NA	China	94.06	99.93	99.61
E. anophelis EA11 (JACLEQ010000009.1)	NA	China	94.38	99.93	99.61
E. anophelis EA12 (JACLER010000009.1)	NA	China	94.38	99.93	99.61
E. anophelis EA13 (JACLES010000006.1)	NA	China	94.05	99.93	99.61

E. anophelis EA14 (JACLET010000006.1)	NA	China	94.05	99.93	99.61
E. anophelis EA15 (JACLEU010000013.1)	NA	China	85.64	99.93	99.03
E. anophelis EA16 (JACLEV010000007.1)	NA	China	94.09	99.93	99.61
E. anophelis EA17 (JACLEW010000007.1)	NA	China	94.05	99.93	100
E. anophelis EA18 (JACLEX010000007.1)	NA	China	93.28	99.74	99.53
E. anophelis EA19 (JACLEY010000007.1)	NA	China	93.28	99.74	99.53
E. anophelis EA20 (JACLFA010000013.1)	NA	China	85.64	99.93	99.03
E. anophelis EA21 (JACLFB010000008.1)	NA	China	93.28	99.74	99.53
E. anophelis EA22 (JACLFC010000013.1)	NA	China	85.63	99.93	99.03
E. anophelis UCLA2018 (JADEZX010000029.1)	Blood	USA	81.68	99.93	99.48
E. anophelis CSID 3000653323 (NWMM01000008.1)	Blood	USA	86.41	99.34	100
E. anophelis CSID 3000726633 (NWMI01000004.1)	Blood	USA	85.7	99.34	99.11
E. anophelis CSID 3000726811 (NWMH01000002.1)	Urine	USA	85.7	99.34	99.11
E. anophelis CSID 3015183680 (MAHP01000007.1)	NA	USA	78.03	99.93	99.48
E. anophelis CSID 3015183686 (MAHR01000042.1)	NA	USA	82.7	99.93	99.48
E. anophelis CSID 3015183678 (MAFY01000020.1)	NA	USA	95.43	99.93	99.69
E. anophelis UCLA EA-1 (JADEZW010000013.1)	Tracheal aspirate	USA	81.68	99.93	99.48
E. anophelis As1 (LFKT01000010.1)	Whole mosquito body	USA	98.18	100	100
E. anophelis F3543 (MAHT01000006.1)	CSF	USA	93.26	99.74	99.53
E. anophelis PHOL-785 (RSAV01000007.1)	Endoscopy specimen	Canada	87.14	99.24	99.16
E. anophelis PHOL-104 (RSAW01000010.1)	Fluid	Canada	94	99.93	99.5
E. anophelis PHOL-537 (RSAX01000013.1)	Urine	Canada	89.01	99.27	99.16
E. anophelis PHOL-515 (RSAY01000011.1)	Blood	Canada	94.53	99.74	99.5

E. anophelis PHOL-090 (RSAZ01000007.1)	Aspirate	Canada	93.75	100	99.29
E. anophelis GX130 (SYWB01000016.1)	Blood	China	94.99	100	99.5
E. anophelis NCTC10586 (UARL01000016.1)	NA	NA	92.86	99.74	99.53
E. anophelis OSUVM1 (PJMA01000002.1)	Equine stall	USA	88.76	99.34	99.11
E. anophelis NCTC10588 (UFYD01000001.1)	NA	NA	94.47	99.93	99.58
E. anophelis 3375 (CP016373.1)	NA	USA	93.29	99.74	99.53
E. anophelis R26 (CP023401.1)	*Anopheles gambiae* G3 adults	Sweden	98.3	100	100
E. anophelis F3543 (CP014340.1)	Cerebrospinal fluid	USA	93.36	99.74	99.53
E. anophelis E6809 (CP014339.1)	Blood	USA	92.59	99.74	99.53
E. anophelis Ag1 (CP023402.1)	Gut	USA	98.29	100	100
E. anophelis 0422 (CP016370.1)	Blood	USA	93.59	99.67	99.56
E. anophelis F3201 (CP016374.1)	NA	Kuwait	89.06	99.27	99.14
E. anophelis FMS-007 (CP006576.1)	Sputum	China	92.31	100	99.5
E. anophelis CSID 3015183684 (CP015066.2)	Blood	USA	94.19	99.93	99.69
E. anophelis CSID 3000521207 (CP015067.2)	NA	USA	94.24	99.93	99.69
E. anophelis CSID 3015183681 (CP015068.2)	NA	USA	94.19	99.93	99.69
E. anophelis SEA01 (CP069277.1)	Blood	India	90.37	98.88	99.27
E. anophelis 2008N07-201 (CP077751.1)	Blood	Taiwan	91.55	100	99.27
E. anophelis 090-MNO-R (CP077749.1)	Blood	Taiwan	94.18	99.67	99.69
E. anophelis 2002C02-176 (CP077754.1)	Respiratory tract	Taiwan	91.54	100	99.27
E. anophelis 229-MNO-R (CP077750.1)	Blood	Taiwan	93.5	100	99.61
E. anophelis 2002N07-090 (CP077753.1)	Blood	Taiwan	94.18	99.67	99.69
E. anophelis 2008S01-229 (CP077752.1)	Blood	Taiwan	93.51	100	99.61

E. anophelis CSID 3015183678 (CP014805.2)	NA	USA	94.19	99.93	99.69
E. anophelis NUHP1 (CP007547.1)	NA	Singapore	93.1	99.93	99.48
E. anophelis JUNP 353 (AP022313.1)	NA	Nepal	93.51	99.93	99.48
E. anophelis 296-96 (CP046080.1)	Blood	Taiwan	94.16	99.93	99.58
E. anophelis AR6-8 (CP023403.1)	NA	China	98.29	100	100
E. anophelis AR4-6 (CP023404.1)	NA	China	98.29	100	100
E. anophelis SUE (CP034247.1)	Blood	Taiwan	94.16	99.93	99.58
E. anophelis FDAARGOS 198 (CP023010.2)	Blood	Sweden	93.28	99.74	99.53
E. anophelis FDAARGOS 132 (CP014020.1)	Endotracheal aspirate	USA	93.84	99.93	99.53
E. anophelis FDAARGOS 134 (CP014021.1)	Endotracheal aspirate	USA	93.84	99.93	99.53
E. anophelis JM-87 (CP016372.1)	NA	USA	87.74	99.34	99.19
E. meningoseptica CCUG 214 (FLSV01000007.1)	NA	NA	98.24	100	100
E. meningoseptica CIP111048 (FTPF01000007.1)	NA	NA	95.35	99.74	99.82
E. meningoseptica SQ101 (JADRJH010000001.1)	NA	China	94.09	99.74	99.82
E. meningoseptica 58-80 CIP80.33 (FTRA01000026.1)	Tracheal exudate	NA	96.08	99.74	99.92
E. meningoseptica NV2016 (FRFB01000010.1)	NA	NA	97.51	99.87	99.87
E. meningoseptica ATCC 13253 (ASAN01000025.1)	NA	NA	98.18	100	100
E. meningoseptica EM1 (MCJH01000009.1)	Blood	NA	96.02	99.74	99.82
E. meningoseptica EM2 (MDTZ01000014.1)	Trachea	USA	95.35	99.8	99.84
E. meningoseptica EM3 (MDTY01000010.1)	Trachea	NA	96.02	99.74	99.82
E. meningoseptica EM4 (JACLGI010000016.1)	NA	China	93.98	99.74	99.82
E. meningoseptica EM5 (JACLGJ010000016.1)	NA	China	94.07	99.74	99.82
E. meningoseptica EM6 (JACLGK010000019.1)	NA	China	94.07	99.74	99.82

E. meningoseptica EM7 (JACLGL010000016.1)	NA	China	94.07	99.74	99.82
E. meningoseptica EM8 (JACLGM010000016.1)	NA	China	94.07	99.74	99.82
E. meningoseptica EM9 (JACLGN010000018.1)	NA	China	94.07	99.74	99.82
E. meningoseptica EM10 (JACLFQ010000014.1)	NA	China	93.62	99.74	99.82
E. meningoseptica EM11 (JACLFR010000017.1)	NA	China	94.07	99.74	99.82
E. meningoseptica EM12 (JACLFS010000017.1)	NA	China	94.07	99.74	99.82
E. meningoseptica EM13 (JACLFT010000017.1)	NA	China	94.07	99.74	99.82
E. meningoseptica EM14 (JACLFU010000017.1)	NA	China	94.07	99.74	99.82
E. meningoseptica EM15 (JACLFV010000017.1)	NA	China	94.07	99.74	99.82
E. meningoseptica EM16 (JACLFW010000016.1)	NA	China	93.62	99.74	99.82
E. meningoseptica EM17 (JACLFX010000016.1)	NA	China	93.62	99.74	99.82
E. meningoseptica EM18 (JACLFY010000009.1)	NA	China	95.44	99.74	99.82
E. meningoseptica EM19 (JACLFZ010000015.1)	NA	China	93.62	99.74	99.82
E. meningoseptica EM20 (JACLGB010000016.1)	NA	China	93.98	99.74	99.82
E. meningoseptica EM21 (JACLGC010000016.1)	NA	China	93.98	99.74	99.82
E. meningoseptica EM22 (JACLGD010000017.1)	NA	China	93.62	99.74	99.82
E. meningoseptica EM23 (JACLGE010000016.1)	NA	China	93.62	99.74	99.82
E. meningoseptica EM24 (JACLGF010000015.1)	NA	China	93.98	99.74	99.82
E. meningoseptica CSID 3000515919 (MAGZ01000024.1)	NA	USA	96.39	99.87	99.74
E. meningoseptica CSID 3000516359 (MAHC01000017.1)	NA	USA	96.09	99.74	99.82
E. meningoseptica CSID 3000516465 (MAHE01000019.1)	NA	USA	95.52	99.8	99.84
E. meningoseptica CSID 3000516535 (MAHF01000018.1)	NA	USA	95.53	99.8	99.82
E. meningoseptica CSID 3000516977 (MAHI01000014.1)	NA	USA	97.54	100	99.87

E. meningoseptica G4076 (MAHZ01000008.1)	NA	United Kingdom	98.24	99.87	100
E. meningoseptica G4120 (MAIA01000008.1)	NA	France	95.51	99.74	99.82
E. meningoseptica 61421 PRCM (MPOG01000006.1)	NA	China	95.41	99.74	99.82
E. meningoseptica GX196 (SYWC01000018.1)	Blood	China	97.58	99.87	99.87
E. meningoseptica NCTC10016 (LS483376.1)	NA	NA	98.28	100	100
E. meningoseptica KC1913 (CP014338.1)	Cerebrospinal fluid	USA	98.3	100	100
E. miricola CIP111047 (FTQX01000011.1)	NA	NA	77.27	100	99.58
E. miricola LDVH-337.01 (CIP108653.1)	Spleen	France	96.07	99.86	99.22
E. miricola IMT47538 (JAAOKX010000023.1)	NA	Germany	96.18	99.86	99.22
E. miricola IMT47318 (JAAOKZ010000034.1)	NA	Germany	95.94	99.86	99.22
E. miricola KCTC 12492 (FLSS01000007.1)	NA	NA	98.3	100	100
E. miricola EM CHUV (LIQC01000021.1)	Endotracheal secretions	Switzerland	72.42	99.93	99.16
E. miricola G4071 (LNOI01000009.1)	Tracheal exudate	France	77.12	100	99.58
E. miricola GTC 862 (LSGQ01000005.1)	NA	NA	98.3	100	100
E. miricola CSID 3000517120 (MAGX01000007.1)	NA	USA	92.58	99.44	99.22
E. miricola CSID 3000516464 (MAHD01000012.1)	NA	USA	78.4	99.51	99.29
E. miricola CSID 3000516998 (MAHK01000017.1)	NA	USA	75.21	99.44	99.61
E. miricola NCTC11305 (UARN01000011.1)	NA	NA	76.88	100	99.58
E. miricola G4074 (MAHY01000011.1)	NA	United Kingdom	77.18	100	99.58
E. miricola Mir-N11 (CP090369.1)	NA	China	93.04	99.86	99.22
E. miricola FL160902 (CP040516.1)	Farming pond	China	93.05	99.86	99.22
E. miricola G4121 (MAIB01000003.1)	NA	Sweden	78.21	100	99.58
E. bruuniana EM798-26 (CP023746.1)	Blood	Taiwan	91.82	99.86	98.98

E. bruuniana BM10 (CP011059.1)	Hindgut	South Korea	81.79	99.51	99.03
E. bruuniana 6012926 (QNTX01000006.1)	Blood	USA	90.23	99.86	99.06
E. bruuniana CSID 3000516589 (MAHG01000002.1)	NA	USA	91.51	99.6	99.48
E. bruuniana CSID 3015183685 (MAHQ01000008.1)	NA	USA	91.67	99.87	99.27
E. bruuniana FDAARGOS 1031 (CP067018.1)	NA	USA	97.7	100	100
E. bruuniana G0153 (MAHW01000018.1)	NA	Ireland	91.22	99.6	99.24
E. bruuniana G0146 (CP014337.1)	NA	United Kingdom	98.3	100	100
E. bruuniana ATCC 33958 (CP035811.1)	Contaminated preparation of carboxypeptidase A	USA	97.57	100	99.97
E. bruuniana G4075 (LNOJ01000023.1)	Blood	France	98.18	100	100
E. ursingii G4122 (LNOK01000023.1)	Soil	Denmark	98.3	100	100
E. ursingii G4123 (CP016377.1)	NA	Denmark	89.78	99.93	99.32
E. ursingii C1558 (MBDS01000012.1)	NA	NA	86.96	99.86	99.4
E. ursingii CSID 3000516135 (MAHB01000010.1)	NA	USA	88.34	100	99.53
E. occulta G4070 (MAHX01000006.1)	NA	Australia	98.3	100	100
E. occulta F8124 (MBDR01000015.1)	NA	NA	97.45	99.6	100
E. argenteiflava YB22 (JAAABJ010000676.1)	Pods	South Korea	98.3	100	100

16S rRNA, 16S ribosomal RNA.

rpoB, RNA polymerase β-subunit.

iDDH, in silico DNA-DNA hybridization.

NA, not available

Table S2. ***Elizabethkingia*** **strains, isolation source, isolation country, and similarity of 16S rRNA gene to** ***E. anophelis*** **subsp.** ***endophytica*** **type strain JM-87 .**

Strain (GenBank accession no.)	Isolation source	Isolation country	16S rRNA similarity (%)
E. anophelis subsp. *endophytica* JM-87 (CP016372.1)	NA	USA	100
E. anophelis 502 (AVCQ01000002.1)	Wound swab	United Kingdom	99.93
E. anophelis B2D (JNCG01000001.1)	Dental plaque	Malaysia	100
E. anophelis CIP111046 (FTRB01000010.1)	NA	NA	99.93
E. anophelis CIP60.58 (FTQY01000009.1)	NA	NA	99.34
E. anophelis CSID 3000516978 (MAHJ01000016.1)	NA	USA	100
E. anophelis CSID 3000517066 (MAHL01000016.1)	NA	USA	100
E. anophelis CSID 3000653323 (NWMM01000008.1)	Blood	USA	100
E. anophelis CSID 3000726633 (NWMI01000004.1)	Blood	USA	100
E. anophelis CSID 3000726811 (NWMH01000002.1)	Urine	USA	100
E. anophelis F3201 (CP016374.1)	NA	Kuwait	99.93
E. anophelis OSUVM1 (PJMA01000002.1)	Equine stall	USA	100
E. anophelis PHOL-537 (RSAX01000013.1)	Urine	Canada	99.93
E. anophelis PHOL-785 (RSAV01000007.1)	Endoscopy specimen	Canada	99.93
E. anophelis PW2809 (CBYE010000030.1)	NA	NA	99.27
E. anophelis PW2810 (CBYF010000021.1)	NA	NA	99.93
E. anophelis SEA01 (CP069277.1)	Blood	India	98.29

NA, not available.

(A) (B)

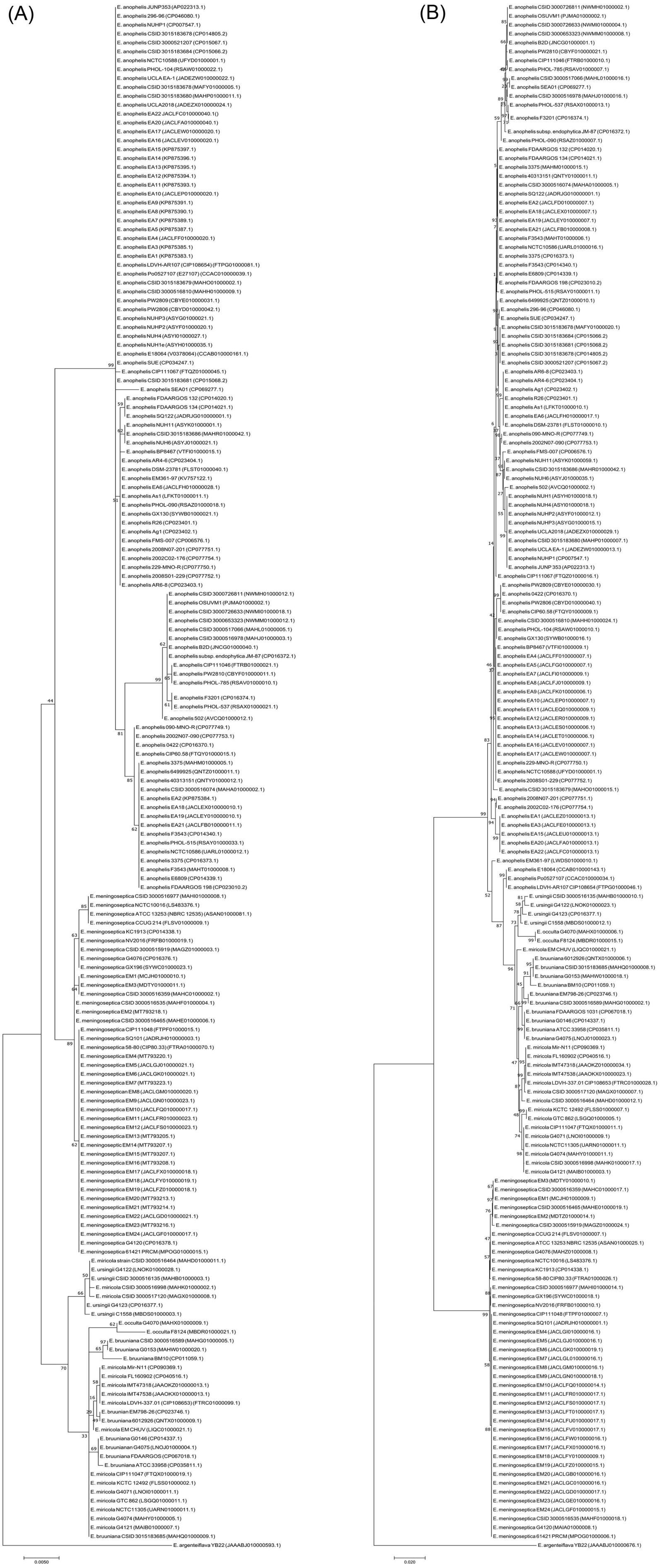

16S rRNA complete *rpoB*

Figure S1. Phylogenetic analysis derived from of the 16S rRNA gene sequences (A) and complete *rpoB* gene sequences (B) in 173 *Elizabethkingia* strains by the maximum-likelihood method based on the Jukes-Cantor model (JC69). The percentage of replicate trees in which the associated taxa clustered together in the bootstrap test of 1000 replicates and branch lengths of the evolutionary distances are shown.
